# Efficacy and safety of zolbetuximab for first-line treatment of advanced Claudin 18. 2-positive gastric or gastro-esophageal junction adenocarcinoma: a systematic review and meta-analysis of randomized controlled trials

**DOI:** 10.3389/fonc.2023.1258347

**Published:** 2023-10-09

**Authors:** Zhanpeng Liang, Liwen Liu, Wenxia Li, Huiqin Lai, Luzhen Li, Jiaming Wu, Huatang Zhang, Cantu Fang

**Affiliations:** Department of Oncology, Zhongshan Hospital of Traditional Chinese Medicine Affiliated to Guangzhou University of Traditional Chinese Medicine, Zhongshan, China

**Keywords:** zolbetuximab, Claudin18.2, gastric or gastro-oesophageal junction adenocarcinoma, targeted therapy, meta-analysis

## Abstract

**Objective:**

Zolbetuximab is a “first-in-class” chimeric lgG1 monoclonal antibody targeting Claudin18.2 (CLDN 18.2). In recent years, several important trials have been published showing that zolbetuximab is associated with improved prognosis in patients with advanced gastric or gastro-esophageal junction (G/GEJ) adenocarcinoma. This promises great change to the current treatment landscape. Therefore, we conducted a systematic review and meta-analysis to evaluate the efficacy and safety of zolbetuximab for first-line treatment of advanced CLDN 18. 2-positive G/GEJ adenocarcinoma.

**Methods:**

The following databases were searched for relevant studies: PubMed, EMBASE, and Cochrane library (updated 10 June 2023). All randomized trials comparing zolbetuximab plus chemotherapy versus first-line chemotherapy alone for first-line treatment of advanced CLDN 18. 2-positive G/GEJ adenocarcinoma were eligible for inclusion. Data were analyzed using Review Manager 5.4.1 (Cochrane collaboration software). Primary outcomes and measures included overall survival (OS), progression-free survival (PFS), objective response rate (ORR), and adverse events (AEs).

**Results:**

This systematic review and meta-analysis included three randomized controlled studies involving 1,402 patients (699 receiving zolbetuximab plus chemotherapy and 703 receiving chemotherapy alone). Compared with chemotherapy alone, zolbetuximab plus chemotherapy significantly improved OS (HR = 0.73; 95% CI: 0.68–0.84) and PFS (HR = 0.64; 95% CI: 0.50–0.82), but did not result in a higher ORR (RR = 0.92; 95% CI: 0.82–1.03). Further analysis of CLDN 18.2 expression showed a more significant benefit for OS (HR = 0.69; 95% CI: 0.55–0.87; *p* = 0.002) and PFS (HR = 0.61; 95% CI: 0.44–0.84; *p* = 0.003) from zolbetuximab in patients with high expression, while there was significant benefit in patients with lower expression. In terms of AEs, zolbetuximab plus chemotherapy was associated with higher risk of grade 3 and higher AEs, but increased risk of nausea and vomiting were more common.

**Conclusion:**

This systematic review and meta-analysis revealed that the effect of zolbetuximab plus chemotherapy was superior to that of chemotherapy alone for first-line treatment of advanced CLDN 18.2-positive G/GEJ adenocarcinoma. Thus, zolbetuximab plus chemotherapy represents a new first-line treatment for these patients. Zolbetuximab plus chemotherapy was associated with higher risk of grade 3 and higher AEs, but was generally manageable.

**Systematic Review Registration:**

https://www.crd.york.ac.uk/prospero, identifier (CRD42023437126).

## 1 Introduction

Gastric and gastro-esophageal junction (G/GEJ) adenocarcinoma is an aggressive form of malignant tumor, and its occurrence has been increasing year-over-year. This not only threatens human health, but also exerts immense financial costs on society. Surgery is a common and effective treatment for resectable G/GEJ adenocarcinoma, but most patients have early local recurrence or distant metastasis after surgery. Advanced metastatic G/GEJ adenocarcinoma is a refractory tumor with poor prognosis, and a median overall survival of 9–14 months (1–5). At present, the first-line standard treatment is guided by three types of molecular characteristics: HER2-positive, HER2-negative, and dMMR/MSI-H. Anti-HER2-targeted therapy and immunotherapy have greatly improved the survival of HER2-positive and PD-L1 highly expressed gastric cancer patients (4, 5). However, it is difficult for HER2-negative patients with low PD-L1 expression to benefit from anti-HER2-targeted therapy and immunotherapy, resulting in its treatment being limited to chemotherapy, which is not an effective way to control the disease (6, 7). Changes in claudin at tight junctions are associated with tight adhesion impairment and epithelial cells’ polarity. These structural abnormalities can lead to increased cell proliferation, epithelial–mesenchymal transformation, invasion, and metastasis (8–10). Despite significant advances in systemic treatment in recent years, the unmet need remains significant. As tumor therapy gradually transitions towards the macromolecular era, target selection for Claudin 18.2 (CLDN 18.2) has become the focus of new drug research and development. Studies have shown that gastric cancers with positive CLDN 18.2 expression (defined as more than 40% of tumor cells with IHC staining intensity ≥2+) account for approximately 49%–85% of gastric cancers (11–13), while gastric cancers with high CLDN 18.2 expression account for approximately 24%–36% of gastric cancers (14, 15). On account of its specificity and high expression in patients with gastric cancer, CLDN 18.2 has become an emerging target for developing new gastric cancer drugs, providing a new direction for targeted gastric cancer therapy. Zolbetuximab is a “first-in-class” chimeric lgG1 monoclonal antibody targeting CLDN 18.2 (16, 17), which is currently being developed for first-line treatment with HER2-negative CLDN 18.2 strongly positive locally advanced unresectable or metastatic G/GEJ adenocarcinoma. Recently, several important trials have been published, showing that first-line treatment with zolbetuximab plus chemotherapy can improve prognosis in patients with advanced G/GEJ adenocarcinoma (18–20). Therefore, we conducted a systematic review and meta-analysis to evaluate the efficacy and safety of zolbetuximab plus chemotherapy for first-line treatment of advanced CLDN 18. 2-positive G/GEJ adenocarcinoma.

## 2 Methods

This study was registered in the PROSPERO database (CRD42023437126) and was conducted according to the preferred reporting project for systematic review and meta-analysis (PRISMA) statement (21). The purpose of this study was to compare the efficacy and safety of zolbetuximab plus chemotherapy and chemotherapy alone for first-line treatment of advanced CLDN 18. 2-positive G/GEJ adenocarcinoma.

### 2.1 Eligibility criteria

The studies were screened independently by two authors. The inclusion criteria for selecting studies in this meta-analysis were as follows: (1) patients with advanced CLDN 18.2-positive G/GEJ adenocarcinoma diagnosed cytologically or pathologically; (2) patients older than 18 years; (3) prospective phase II or III, randomized clinical trials evaluating the efficacy and safety of zolbetuximab; and (4) studies reporting at least one of the following outcomes: overall survival (OS), progression-free survival (PFS), objective response rate (ORR), and adverse events (AEs). CLDN 18.2 positivity was defined as moderate (2+) or strong CLDN18.2 staining (3+) in ≥40% of tumor cells. CLDN 18.2 high expression was defined as moderate (2+) or strong CLDN18.2 staining (3+) in ≥70% of tumor cells.

Exclusion criteria were as follows: (1) patients with early G/GEJ adenocarcinoma; (2) non-randomized controlled studies, basic studies, retrospective studies, case reports, duplicate publications, and studies for which no relevant data could be extracted; and (3) randomized controlled trials (RCTs) that were based on overlapping patients.

### 2.2 Search strategy

RCTs evaluating the efficacy and safety of zolbetuximab for first-line treatment of advanced CLDN 18. 2-positive G/GEJ adenocarcinoma were identified by a computerized search of PubMed, Embase, and Cochrane Library, using the following search terms: gastric cancer, gastro-esophageal adenocarcinoma, zolbetuximab, claudin 18.2, and IMAB362. The relevant bibliography of candidate articles was manually searched to identify additional studies. The proceedings of the American Society of Clinical Oncology (ASCO) and the European Society of Medical Oncology (ESMO)/European Cancer Congress (ECC) annual meetings were searched for abstract reports of relevant studies. If there was any overlapping data, the most complete and updated report was selected for inclusion in this meta-analysis. Additionally, the references from all eligible studies were manually reviewed to identify any other relevant studies.

### 2.3 Study selection and data extraction

Two experienced investigators independently screened the records for eligibility. Differences were resolved by consulting a third investigator. Titles and abstracts were browsed to complete an initial selection, followed by a full review of potentially eligible articles and the selection of eligible articles based on pre-established criteria.

Extracted data included baseline characteristics, sample size and interventions used, number of assessable patients, PFS, OS, ORR, grade 3, and higher AEs. Two investigators independently extracted relevant data and resolved any differences by consulting a third investigator. When multiple articles contained overlapping patient series, we prioritized the extraction of outcome data from the primary articles with the largest sample size for early outcomes and the articles with the longest follow-up duration for the late outcomes.

### 2.4 Outcome

The results of this review include OS, PFS, ORR, and AEs. OS is defined as the time from randomization to death. PFS is defined as the time from randomization to death or disease progression, whichever occurs first. ORR reflects the proportion of patients with complete response and partial response. AEs, graded according to National Cancer Institute Common Terminology Criteria for Adverse Events version 4.03, included all grades of AEs and grade 3 or higher AEs.

### 2.5 Risk of bias

Two investigators independently assessed the quality of the included trials using the Cochrane Collaboration tools with respect to randomized sequence generation, assignment concealment, blinding, incomplete outcome data, and selective outcome reporting (22). Any differences in quality assessment were resolved by consulting a third investigator.

### 2.6 Statistical analysis

Data were analyzed using Review Manager 5.4.1 (Cochrane Collaboration Software). These measures were either extracted directly from the articles or calculated. ORR and AEs were reported as risk ratio (RR) with corresponding 95% confidence intervals (95% CI). PFS and OS were reported as hazard ratio (HR) and had 95% CI. *p* < 0.05 was considered statistically significant. For effectiveness or side effects, HR or RR > 1 favored chemotherapy alone (control), while HR or RR < 1 favored zolbetuximab plus chemotherapy (experimental). Heterogeneity was tested with an *I*² statistic. Unless heterogeneity was high, in which case a random-effects model was used, a fixed-effects model is used for data synthesis (23, 24). Funnel plots and an Egger test were adopted to investigate the potential for publication bias (25). Subgroup analysis was conducted for age, sex, region, previous gastric cancer surgery, Lauren classification, tumor location, and number of metastatic sites.

## 3 Results

### 3.1 Study identification and quality assessment

**Figure 1:**
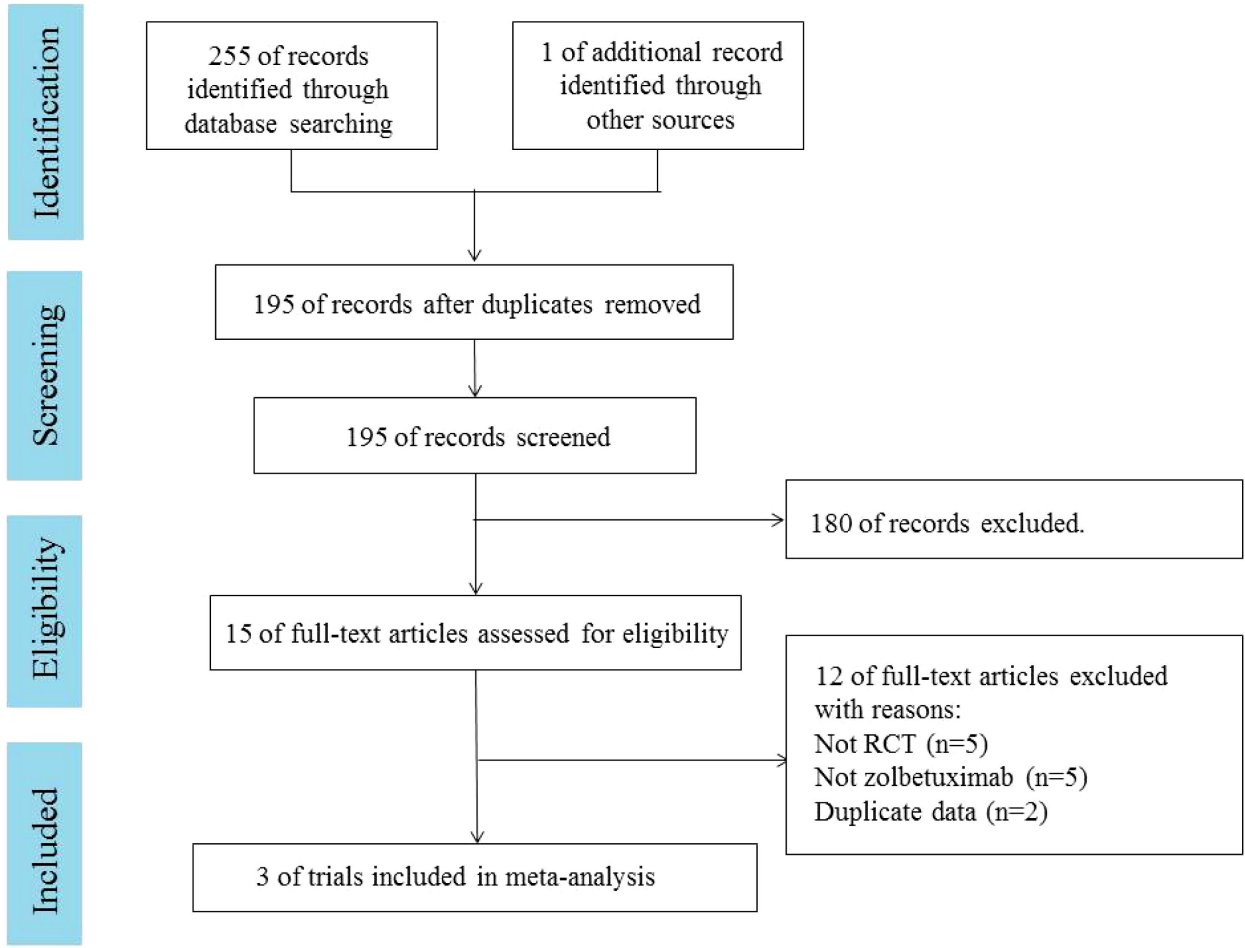
PRISMA flow diagram. RCT, randomized controlled trial.

A total of 255 articles were retrieved from PubMed, EMBASE, and the Cochrane Library. One additional article was retrieved from ASCO. Duplicates were excluded in 61 cases, and 180 cases were excluded by reading the title and abstract. Fifteen articles were read in full. Three RCTs (18–20), involving 1,402 patients, were included. A PRISMA flow chart describing study identification and selection is shown in **Figure 1**. Since all studies included were randomized, selection and loss bias were minimized. In one trial (18), blinding was not applied, which could have resulted in some bias.

### 3.2 Study and patient characteristics

FAST (18) was an open-label, randomized controlled, phase II clinical trial that enrolled 252 eligible patients between July 2012 and June 2014. SPOTLIGHT (19) is a multicenter, randomized, double-blind, phase III trial that enrolled 565 eligible patients between June 2018 and April 2022. GLOW (20) is also a multicenter, randomized, double-blind, phase III trial that enrolled 507 eligible patients between November 2018 and February 2022.

All three trials evaluated the prognostic effect of zolbetuximab plus chemotherapy as a first-line treatment for HER2-negative, CLDN 18.2-positive, locally advanced, unresectable or metastatic G/GEJ adenocarcinoma. However, the chemotherapy regimens differed among the three trials. The EOX regimen (epirubicin, oxaliplatin, and capecitabine) was used in FAST (18). In SPOTLIGHT (19), patients received chemotherapy with the mFOLFOX6 regimen (modified folinic acid [or levofolinate], fluorouracil, and oxaliplatin regimen). Patients were treated with chemotherapy with the CAPOX regimen (oxaliplatin and capecitabine) in GLOW (20).

FAST (18) evaluated two different doses of zolbetuximab. One was administered at a loading dose of 800 mg/m^2^ in Cycle 1 followed by 600 mg/m^2^ in subsequent cycles, which was the same as that used in SPOTLIGHT (19) and GLOW (20). The other was administered at 1,000 mg/m^2^ per cycle. All three trials included patients with strong CLDN 18.2 positivity, with similar, but non-identical, definitions. The FAST (18) study enrolled advanced G/GEJ adenocarcinoma patients with moderate-to-strong CLDN18.2 expression in ≥40% tumor cells. SPOTLIGHT (19) and GLOW (20) enrolled advanced G/GEJ adenocarcinoma patients with moderate-to-strong CLDN 18.2 expression in ≥75% tumor cells. The baseline characteristics of the patients included in the study are detailed in **Table 1**.

**Table 1:** Characteristics of included studies and patients.

	FAST			SPOTLIGHT		GLOW	
	Zolbetuximab (800/600 mg/m²)	Zolbetuximab (1,000 mg/m²)	Control	Zolbetuximab	Control	Zolbetuximab	Control
**Key eligibility criteria**	Moderate-to-strong CLDN18.2 expression in ≥40% tumor cells			Moderate-to-strong CLDN18.2 expression in ≥75% tumor cells		Moderate-to-strong CLDN18.2 expression in ≥75% tumor cells	
**Schedule**	Zolbetuximab (loading dose, 800 mg/m^2^ then 600 mg/m^2^ Q3W) + EOX	Zolbetuximab (1,000 mg/m^2^ Q3W) + EOX	EOX	Zolbetuximab (loading dose, 800 mg/m^2^ then 600 mg/m^2^ Q3W) + mFOLFOX6	Placebo + mFOLFOX6	Zolbetuximab (loading dose, 800 mg/m^2^ then 600 mg/m^2^ Q3W) + CAPOX	Placebo + CAPOX
**Patients randomized**	77	85	84	283	282	254	253
**Sex** **Male** **Female**	47 (61%) 30 (39%)	57 (67%) 28 (33%)	56 (67%) 28 (33%)	176 (62%) 107 (38%)	175 (62%) 107 (38%)	159 (63%) 95 (37%)	156 (62%) 97 (38%)
**Median age (range)**	59 (22−77)	60 (28−77)	57 (24−73)	62 (NA)	60 (NA)	61 (22–82)	59 (21–83)
**Region** **Asia** **Non-Asia**	NA	NA	NA	88 (31%) 195 (69%)	89 (32%) 193 (68%)	157 (62%) 97 (38%)	158 (63%) 95 (37%)
**Tumor site** **Stomach** **GEJ**	62 (81%) 15 (19%)	77 (91%) 8 (9%)	68 (81%) 16 (19%)	219 (77%) 64 (23%)	210 (74%) 72 (26%)	219 (86%) 35 (14%)	209 (83%) 44 (17%)
**ECOG** **0** **1** **2** **Missing**	23 (30%) 54 (70%) 0 0	27 (32%) 58 (68%) 0 0	25 (30%) 59 (70%) 0 0	125 (44%) 153 (54%) 1 (<1%) 4 (1%)	115 (41%) 163 (58%) 0 4 (1%)	108 (43%) 145 (57%) 0 1 (<1%)	108 (43%) 142 (57%) 0 1 (<1%)
**Lauren classification** **Diffuse** **Intestinal** **Mixed/Unknown/Other** **Missing**	35 (45%) 26 (34%) 16 (21%) 0	39 (46%) 23 (27%) 23 (17%) 0	38 (45%) 27 (32%) 19 (23%) 0	82 (29%) 70 (25%) 130 (46%) 1 (<1%)	117 (41%) 66 (23%) 95 (35%) 4 (1%)	87 (34%) 36 (14%) 130 (51%) 1 (<1%)	100 (40%) 41 (16%) 112 (44%) 0
**Organs with metastases** **0**–**2** **3**	NA	NA	NA	219 (77%) 64 (23%)	219 (78%) 63 (22%)	189 (74%) 65 (26%)	188 (74%) 65 (26%)
**Previous gastrectomy** **Yes** **No**	21 (27%) 56 (73%)	24 (28%) 81 (72%)	23 (27%) 61 (73%)	84 (30%) 199 (70%)	82 (29%) 200 (71%)	179 (70%) 75 (30%)	178 (70%) 75 (30%)

EOX, epirubicin, oxaliplatin, and capecitabine; mFOLFOX6, folinic acid (or levofolinate optionally in Japan), fluorouracil, and oxaliplatin; CAPOX, capecitabine and oxaliplatin; NA, not available; ECOG, performance status score; GEJ, gastro-esophageal junction.

#### 3.3.1 Overall survival

**Figure 2:**
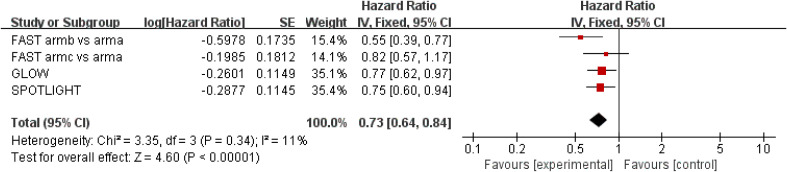
Assessment of overall survival. The diamond indicates best estimate of the true (pooled) outcome (with width indicating 95% CI); HR, hazard ratio; experimental stands for zolbetuximab plus chemotherapy; control stands for chemotherapy alone. Since there is low heterogeneity, a fixed-effects model is used.

Results for OS came from three studies (18–20) involving a total of 1,402 patients. The results showed that zolbetuximab plus chemotherapy further increased OS and reduced the risk of death by 27% (HR = 0.73; 95% CI: 0.68–0.84; *p* < 0.00001) (**Figure 2**). Additionally, low heterogeneity was found among the trials (*χ* ^2^ = 3.35; df = 3 [*p* = 0.34]; *I* ^2^ = 11%). No significant benefit was found in the high-dose study, but the results still favored zolbetuximab plus chemotherapy after excluding the high-dose study (HR = 0.72; 95% CI: 0.62–0.83; *p* < 0.00001) (**eFigure 1**). Further analysis of CLDN 18.2 expression revealed that zolbetuximab plus chemotherapy was associated with significant OS benefit in patients with high expression, reducing the risk of death by 31% (HR = 0.69; 95% CI: 0.55–0.87; *p* = 0.002), but no significant benefit was found in patients with lower expression (**eFigure 2**).

#### 3.3.2 Progression-free survival

**Figure 3:**
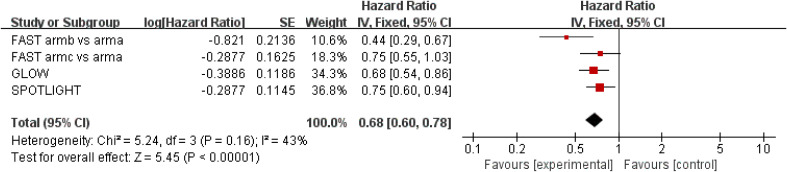
Assessment of progression-free survival. The diamond indicates best estimate of the true (pooled) outcome (with width indicating 95% CI); HR, hazard ratio; experimental stands for zolbetuximab plus chemotherapy; control stands for chemotherapy alone. Since there is moderate heterogeneity, a fixed-effects model is used.

Results for PFS were extracted from three studies (18–20), which included a total of 1,400 patients. Zolbetuximab plus chemotherapy was associated with higher PFS (HR = 0.68; 95% CI: 0.60–0.78; *p* < 0.00001), and it reduced the risk of disease progression by 32%. Moderate heterogeneity was found among the trials (*χ* ^2^ = 5.24; df = 3 [*p* = 0.16]; *I* ^2^ = 43%) (**Figure 3**). Significant benefit was found in the high-dose study, and the results still favored zolbetuximab plus chemotherapy after excluding the high-dose study (HR = 0.64; 95% CI: 0.50–0.82; *p* = 0.0005) (**eFigure 3**). Further analysis of CLDN 18.2 expression showed that zolbetuximab plus chemotherapy was associated with a significant PFS benefit in patients with high expression, reducing the risk of death by 39% (HR = 0.61; 95% CI: 0.44–0.84; *p* = 0.003), but no significant benefit was found in patients with lower expression (**eFigure 4**).

#### 3.3.3 Objective response rate

**Figure 4:**
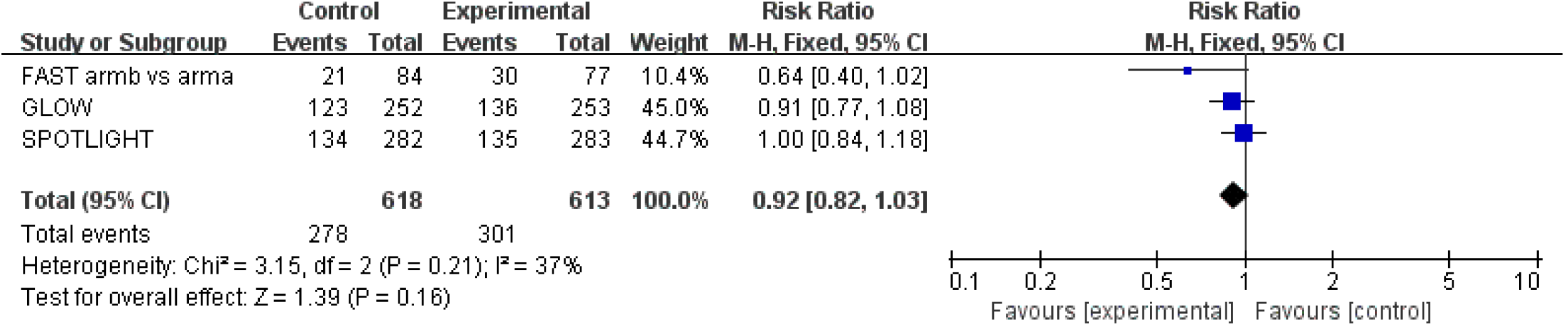
Assessment of objective response rate. The diamond indicates best estimate of the true (pooled) outcome (with width indicating 95% CI); RR, risk ratio; experimental stands for zolbetuximab plus chemotherapy; control stands for chemotherapy alone. Since there is moderate heterogeneity, a fixed-effects model is used.

ORR results were extracted from three studies (18–20) involving a total of 1,231 patients. Zolbetuximab plus chemotherapy did not result in a higher ORR (RR = 0.92; 95% CI: 0.82–1.03; *p* = 0.016) (**Figure 4**). Moderate heterogeneity was found among the trials (*χ* ^2^ = 3.15; df = 2 [*p* = 0.21]; *I* ^2^ = 37%).

#### 3.3.4 Adverse events

Data on AEs were extracted from three studies (18–20) involving 1,394 patients. In terms of AEs of all grades, there was no statistical difference between zolbetuximab plus chemotherapy and chemotherapy alone due to the higher incidence of AEs (**Table 2**). In all of grade AEs by preferred terms, zolbetuximab plus chemotherapy was associated with a higher incidence of nausea, vomiting, neutropenia, decreased appetite, and peripheral edema. On the other hand, zolbetuximab plus chemotherapy resulted in a higher risk of grade 3 and higher AEs, including nausea, vomiting, neutropenia, decreased appetite, and weight loss. Further analysis showed that zolbetuximab plus chemotherapy significantly increased nausea and vomiting in patients who did not undergo gastrectomy compared with chemotherapy alone. In patients who had undergone gastrectomy, zolbetuximab plus chemotherapy increased vomiting, but not nausea (**eFigures 5**, **6**). However, owing to the small amount of data included, the data on AEs were not yet mature.

**Table 2:** Results of adverse events.

Toxicity	All grade AEs (risk ratio)	No. of trials	Grade 3+ AEs (risk ratio)	No. of trials
Any adverse event	1.00 [0.98 1.01]	4	1.08 [1.02, 1.15]	3
Gastrointestinal disorders				
Nausea	1.23 [1.09, 1.39]	4	2.59 [1.70, 3.94]	3
Vomiting	1.66 [1.32, 2.10]	4	3.02 [2.00, 4.56]	3
Diarrhea	0.79 [0.55, 1.14]	4	1.01 [0.61, 1.65]	3
Hematologic disorders				
Anemia	1.07 [0.91, 1.26]	4	1.01 [0.72, 1.41]	3
Neutropenia	1.22 [1.04, 1.43]	4	1.39 [1.09, 1.76]	3
Thrombocytopenia	0.91 [0.62, 1.33]	4	0.80 [0.37, 1.73]	3
Metabolism and nutrition disorder				
Weight loss	1.20 [0.87, 1.65]	4	2.62 [1.04, 6.62]	3
Increased ALT	0.73 [0.49, 1.09]	4	0.41 [0.16, 1.03]	3
Increased AST	0.91 [0.73, 1.13]	4	0.81 [0.38, 1.70]	3
Increased GGT	1.47 [0.73, 2.98]	2	Not estimable	2
Nervous system disorders				
Paresthesia	1.04 [0.76, 1.43]	3	Not estimable	
Headache	0.81 [0.59, 1.12]	3	0.74 [0.14, 3.81]	2
General disorders				
Fatigue	1.00 [0.76, 1.30]	4	1.13 [0.67, 1.89]	3
Asthenia	1.08 [0.88, 1.34]	4	1.07 [0.67, 1.71]	3
Other disorders				
Decreased appetite	1.23 [1.01, 1.48]	4	2.17 [1.20, 3.93]	3
Upper abdominal pain	0.68 [0.27, 1.72]	3	1.93 [0.08, 45.83]	2
Abdominal pain	0.83 [0.68, 1.02]	4	1.18 [0.55, 2.52]	3
Alopecia	1.34 [0.91, 1.99]	2	Not estimable	
Pyrexia	0.92 [0.59, 1.43]	4	1.66 [0.22, 12.53]	2
Peripheral edema	2.14 [1.52, 3.01]	4	4.98 [0.24, 103.31]	2
Palmar–plantar syndrome	0.93 [0.54, 1.62]	3	Not estimable	

ALT, alanine aminotransferase; AST, aspartate aminotransferase; GGT, gamma-glutamyl transferase.

### 3.4 Subgroup analysis of patients with CLDN 18.2 high expression

Overall, we found differences in subgroup analysis of age, region, number of metastatic sites, primary sites, and Lauren classification. However, no differences were observed in subgroup analyses of sex or previous gastrectomy. In the ≤65 years old, Asian, 0–2 metastatic sites, stomach, diffuse, and intestinal subgroups, zolbetuximab significantly improved OS and PFS. However, in the > 65 years old, non-Asian, ≥3 metastatic sites, GEJ, and mixed or other subgroups, zolbetuximab did not lead to higher OS or PFS (**Table 3** and **eFigures 7**, **8**).

**Table 3:** Results of subgroup analysis.

Subgroup	Overall survival (HR)	No. of trials	*p*-value	Progression-free survival (HR)	No. of trials	*p*-value
Age						
≤65	0.70 [0.58, 0.85]	2	0.0003	0.68 [0.54, 0.86]	2	0.001
>65	0.84 [0.64, 1.10]	2	0.20	0.79 [0.60, 1.05]	2	0.11
Sex						
Male	0.77 [0.63, 0.93]	2	0.008	0.73 [0.60, 0.89]	2	0.002
Female	0.73 [0.56, 0.95]	2	0.02	0.71 [0.54, 0.92]	2	0.01
Region						
Asia	0.66 [0.53, 0.83]	2	0.0004	0.58 [0.45, 0.73]	2	<0.00001
Non-Asia	0.84 [0.67, 1.03]	2	0.10	0.88 [0.71, 1.09]	2	0.23
Number of metastatic sites						
0–2	0.74 [0.61, 0.89]	2	0.001	0.71 [0.59, 0.86]	2	0.0003
≥3	0.78 [0.58, 1.05]	2	0.10	0.76 [0.56, 1.02]	2	0.07
Primary site						
Stomach	0.67 [0.57, 0.78]	3	<0.00001	0.61 [0.50, 0.75]	3	<0.00001
GEJ	0.90 [0.57, 1.44]	3	0.67	0.99 [0.57, 1.72]	3	0.97
Lauren classification						
Diffuse	0.61 [0.40, 0.94]	3	0.02	0.57 [0.36, 0.89]	3	0.01
Intestinal	0.64 [0.47, 0.87]	3	0.005	0.60 [0.44, 0.83]	3	0.002
Mixed or other	0.74 [0.41, 1.33]	3	0.31	0.79 [0.55, 1.15]	3	0.22
Previous gastrectomy						
No	0.50 [0.35, 0.73]	3	0.0003	0.56 [0.38, 0.83]	3	0.004
Yes	0.82 [0.69, 0.98]	3	0.003	0.74 [0.61, 0.89]	3	0.001

HR, Hazard ratio; GEJ, Gastro-esophageal junction.

### 3.5 Sensitivity analyses and publication bias

Sensitivity analysis via study-by-study removal showed that no study affected the overall effect of the efficacy and safety endpoints, meaning that all of the results were stable. Qualitative assessment was performed by assessing various measures for each individual study using the Cochrane Risk of Bias Tool. Overall, these trials were considered to have low risk of bias. The main source of bias was the lack of blinding in one study (18). Funnel plot asymmetry is not obvious to any efficacy endpoints (**eFigures 9**–**11**). Egger regression test results showed that OS (*p* = 0.579), PFS (*p* = 0.233), and ORR (*p* = 0.243) had a low potential for publication bias.

## 4 Discussion

In unresectable G/GEJ adenocarcinoma, first-line treatment consists of chemotherapy plus either immunotherapy for HER2-negative CPS-PDL1-positive (≥5) tumors (5) or trastuzumab for HER2-positive disease (4). However, the prognosis for HER2-negative and CPS-PDL1 positive (<5) advanced gastric cancer patients treated mainly by chemotherapy is still not optimistic. This indicates an urgent need for new and more efficient therapies for advanced gastric cancer indications in the clinic. CLDN 18.2 is a membrane protein involved in maintaining intercellular adhesion and connection. It has two subtypes: Claudin 18.1 and CLDN 18.2. The former is mainly expressed in normal lung cells, while the latter is only expressed in the differentiated epithelial cells of gastric mucosa (26). Jovov et al. (27) recently described how CLDN18.2 is activated during the metaplastic transition from the stratified squamous cell epithelium of the esophagus to the specialized columnar epithelium. This occurs in the context of gastro-esophageal reflux and predisposes subjects to distal esophageal adenocarcinoma, suggesting that ectopic activation of CLDN 18.2 may be an early event of esophageal adenocarcinoma. Moreover, various Claudins in human cancers have a wide range of expression patterns. CLDN3, 4, and 7 are highly expressed in most normal epithelial cells and their corresponding tumors (28). In contrast to CLDN 18.2, these claudins are widely expressed in healthy tissues. Therefore, therapy targeting of these claudins inevitably leads to significant toxicity. In contrast, other studies have shown that CLDN 18.2 is absent in the stem cell region of gastric cancer, but its exclusive expression in differentiated gastric cells, combined with transient gastrointestinal toxicity, is a common and manageable adverse event (13), making this molecule an effective drug target for G/GEJ adenocarcinoma. Zolbetuximab is highly selective against CLDN 18.2, both *in vivo* and *in vitro*. It binds to cancer-specific targets expressed primarily in tumor cells, and mediates tumor cell death through antibody-dependent cell-mediated cytotoxicity (ADCC) and complement-dependent cytotoxicity (CDC) (29). Recent clinical trials have shown that zolbetuximab is associated with significant improvement in the prognosis of patients with advanced G/GEJ cancer. This verifies the druggability of the CLDN 18.2 target (18–20). Therefore, we performed a meta-analysis to evaluate the efficacy and safety of zolbetuximab in advanced CLDN 18. 2-positive G/GEJ adenocarcinoma. The pooled results showed that zolbetuximab plus chemotherapy for first-line treatment significantly improved PFS and OS in patients with advanced unresectable G/GEJ adenocarcinoma compared to chemotherapy alone.

A phase I study in Japan evaluating zolbetuximab monotherapy in previously treated Japanese patients with CLDN 18.2-positive locally advanced G/GEJ adenocarcinoma showed that 11 of 17 patients achieved stable disease (30). MONO, a phase II study, showed that zolbetuximab monotherapy in recurrent/refractory CLDN 18.2-positive gastric cancer had an ORR of 9% and a clinical benefit rate of 23% (31). The finding that the single drug zolbetuximab has certain anti-tumor activities is not novel. Preclinical studies found that chemotherapy agents upregulated CLDN 18.2 expression and enhance zolbetuximab-induced ADCC (17, 29). These results suggest that zolbetuximab combined with chemotherapy may have superior efficacy. Additionally, the ILUSTRO trial showed that zolbetuximab plus mFOLFOX6 for first-line treatment showed positive results (32). These data support further development of zolbetuximab as a first-line treatment. FAST is the first RCT to evaluate the efficacy of zolbetuximab, compared to zolbetuximab plus EOX and EOX alone. OS and PFS showed significant improvement in the combined treatment group, indicating that zolbetuximab may be an effective supplement to chemotherapy (18). Stratified analysis of CLDN 18.2 expression intensity showed that patients with high CLDN 18.2 expression benefited more from zolbetuximab, but patients with lower CLDN 18.2 expression did not benefit. Therefore, two phase III trials, SPOTLIGHT and GLOW, only included patients with advanced G/GEJ cancer with high CLDN 18.2 expression (19, 20). Our meta-analysis also stratified CLDN 18.2 expression intensity, and the results were consistent with those of the FAST trial. However, since only one study reported survival data in patients with lower CLDN 18.2 expression, further clinical trials are needed to explore CLDN 18.2 expression’s effect on zolbetuximab efficacy. On the other hand, FAST evaluated two different doses of zolbetuximab. Interestingly, high doses of zolbetuximab did not improve survival in CLDN 18.2-positive patients with advanced gastric cancer (18). Our meta-analysis showed that the pooled results were still favorable for zolbetuximab after excluding studies with high doses of zolbetuximab, possibly because high doses of zolbetuximab led to higher discontinuation rates and reduced treatment duration, thus curbing its efficacy. The included studies used different chemotherapy regimens, but they were all approved for first-line treatment of gastric cancer, and their benefits in first-line gastric cancer treatment were similar. In addition, baseline characteristics were balanced in both groups of patients enrolled in the trial. Notably, zolbetuximab plus chemotherapy reduced the risk of death similarly in the SPOTLIGHT (19) and GLOW (20) trials, and low heterogeneity was observed in the outcomes of PFS and OS in our study. Therefore, different chemotherapy regimens have little effect on the efficacy of zolbetuximab. On the other hand, chemotherapy duration does affect zolbetuximab efficacy. Since chemotherapy can boost zolbetuximab’s effectiveness, the longer the chemotherapy treatment duration, the more effective zolbetuximab may be. The median exposure times for chemotherapy in the three trials included in our study were similar (18–20), and thus, the difference in this effect was small.

In the analysis of 523 cases of G/GEJ cancer tissue samples, COATI et al. (33) found that the difference in CLDN 18.2 expression was related to tumor location, Lauren classification, and Epstein–Barr virus infection. In addition, studies (14, 34) have shown that the CLDN 18.2 expression is also correlated with age, tumor stage, peritoneal metastasis, and liver metastasis. In contrast, other studies (11, 33) have shown that CLDN 18.2 expression is not associated with race, age, sex, or tumor stage. To further explore baseline characteristics’ effects on zolbetuximab efficacy, we performed a subgroup analysis. In the ≤65 years old, Asian, 0–2 metastatic sites, stomach, diffuse, and intestinal subgroups, zolbetuximab plus chemotherapy significantly improved OS and PFS. However, in the > 65 years old, non-Asian, ≥3 metastatic sites, GEJ, and mixed or other subgroups, zolbetuximab did not lead to higher OS or PFS. Our meta-analysis indicated that zolbetuximab’s efficacy appeared to be correlated with age, region, number of metastatic sites, primary sites, and Lauren classification. It is worth noting that the results of subgroup analysis should be interpreted with caution because the subgroup analysis data are still immature.

For AEs, owing to the numerous side effects of chemotherapy, the incidence of adverse events of all grades for chemotherapy alone and zolbetuximab plus chemotherapy were high, and there was no statistical difference between the two groups. Thus, it was difficult to evaluate zolbetuximab’s safety. Therefore, we conducted a summary analysis of grade 3 and higher AEs. Zolbetuximab plus chemotherapy were found to be associated with higher risk of grade 3 and higher AEs, but mainly with an increased risk of nausea and vomiting, which can be alleviated with preventative drugs and with treatment. Patients in the combination treatment group were associated with longer drug treatment duration, leading to longer exposure to chemotherapy, which may have contributed to the increased risk of nausea and vomiting. Overall, the adverse effects of zolbetuximab were manageable. Further analysis showed that zolbetuximab plus chemotherapy significantly increased nausea and vomiting in patients who had not undergone gastrectomy compared with chemotherapy alone. In patients with previous gastrectomy, zolbetuximab plus chemotherapy increased the incidence of vomiting, but not nausea. Looking at incidence alone, in the three included trials, patients treated with zolbetuximab who had not undergone gastrectomy had a higher incidence of nausea and vomiting than patients who had previously undergone gastrectomy (18–20). Target-specific organ toxicity based on a drug-related pharmacodynamic mechanism, a higher antigen load in the stomach with the primary tumor still present, or the absence of an intact stomach as an effector organ for vomiting may be explanations for this (31). In FAST (18), no treatment-related fatal AEs occurred, and in SPOTLIGHT, five and four treatment-related fatal AEs were reported in the zolbetuximab plus chemotherapy and chemotherapy alone groups, respectively (19). In addition, treatment-related fatal AEs in the GLOW trial were reported in six and seven cases in the zolbetuximab plus chemotherapy and chemotherapy alone groups, respectively (20). Treatment-related fatal AEs were not statistically different between the two groups.

As a target that has attracted much attention from the global industry in recent years, CLDN 18.2 has been shown to be expressed in various cancer types, including gastric, pancreatic, and esophageal cancer (13). Although zolbetuximab is the first monoclonal antibody to target CLDN 18.2, a major limitation of its efficacy is that it can only be used in patients with high Claudin18.2 expression and is very limited in patients with low CLDN 18.2 expression. Osemitamab (TST001) is a monoclonal antibody with a higher affinity for CLDN 18.2 (35). ASCO recently published a prospective phase II clinical study of Osemitamab to explore the safety and efficacy of TST001 in combination with capecitabine and oxaliplatin (CAPOX) as a first-line treatment for advanced G/GEJ cancer. A total of 42 patients had measurable lesions, of which 28 (66.7%) achieved a partial response (36). Of note, G/GEJ cancer patients with low CLDN18.2 expression (≥10% of tumor cells with CLDN18.2 membrane staining intensity ≥1+) still benefitted from Osemitamab. However, this was a phase II clinical study with a small sample size, and more large RCT s are needed for further verification. In SPOTLIGHT, patients showed significant improvements in OS and PFS regardless of the PD-L1 expression level (19). The combination of anti-CLDN 18.2 drugs and anti-PD-1 drugs may also become a new therapeutic direction. An ongoing phase II study (ILUSTRO) is evaluating zolbetuximab in combination with nivolumab for first-line treatment of gastric cancer. It is expected that the results will provide a meaningful reference for clinical practice.

Our meta-analysis has some limitations. First, we only included a small number of trials. Second, in one trial, blindness was not used, which may have introduced some bias. Third, there were insufficient data to assess zolbetuximab’s efficacy in patients with lower CLDN18.2 expression. Thus, the benefit of zolbetuximab was still limited to patients with high CLDN18.2 expression. Fourth, we did not have access to individual data for logistic regression to adjust the variables such as age, tumor site, previous gastrectomy, etc.

## 5 Conclusion

Our meta-analysis showed that zolbetuximab plus chemotherapy for first-line treatment significantly improved PFS and OS in patients with advanced CLDN 18.2-positive G/GEJ adenocarcinoma compared to using chemotherapy alone. Patients with high CLDN 18.2 expression were more likely to benefit from additional zolbetuximab. Zolbetuximab was associated with higher risk of grade 3 and higher AEs, but mainly with an increased risk of nausea and vomiting, which can be alleviated with drug prevention and treatment. Additional studies are needed to evaluate the effect of CLDN 18.2 expression and baseline characteristics on zolbetuximab’s efficacy.

## Data availability statement

The original contributions presented in the study are included in the article/**Supplementary Material**. Further inquiries can be directed to the corresponding author.

## Author contributions

ZL: Conceptualization, Data curation, Formal Analysis, Investigation, Methodology, Project administration, Software, Writing – original draft. LWL: Conceptualization, Data curation, Formal Analysis, Funding acquisition, Investigation, Methodology, Software, Writing – original draft. WL: Formal Analysis, Methodology, Software, Writing – review & editing. HL: Data curation, Formal Analysis, Methodology, Software, Writing – review & editing. LZL: Resources, Supervision, Validation, Visualization, Writing – review & editing. JW: Resources, Supervision, Validation, Visualization, Writing – review & editing. HZ: Resources, Supervision, Validation, Visualization, Writing – review & editing. CF: Conceptualization, Funding acquisition, Project administration, Resources, Software, Supervision, Validation, Visualization, Writing – review & editing.
