# Supplementary material for: Efficacy and safety of zolbetuximab for first-line treatment of advanced Claudin 18. 2-positive gastric or gastro-esophageal junction adenocarcinoma: a systematic review and meta-analysis of randomized controlled trials
Source: Front Oncol. 2023 Oct 9;13:1258347. doi: 10.3389/fonc.2023.1258347 (PMC10598679; doi:10.3389/fonc.2023.1258347)
Supplement: Supplementary file 1 [file DataSheet_1.docx]

Supplementary

Context:

**eFigure. 1** Results of subgroup analysis of zolbetuximab dose for overall survival.

**eFigure. 2** Results of subgroup analysis of CLDN 18.2 expression for overall survival.

**eFigure. 3** Results of subgroup analysis of zolbetuximab dose for progression-free survival

**eFigure. 4** Results of subgroup analysis of patients with CLDN 18.2 high expression for progression-free survival

**eFigure. 5** Results for the effect of previous gastrectomy surgery in nausea

**eFigure. 6** Results for the effect of previous gastrectomy surgery in voting

**eFigure. 7** Results of subgroup analysis of patients with CLDN 18.2 high expression for overall survival.

**eFigure. 8** Results of subgroup analysis of patients with CLDN 18.2 high expression for progression-free survival

**eFigure. 9** Funnel plot of overall survival.

**eFigure. 10** Funnel plot of progression free survival.

**eFigure. 11** Funnel plot of objective response rate

**eMethods.** Search Strategy

**eFigure. 1** Results of subgroup analysis of zolbetuximab dose for overall survival.

**
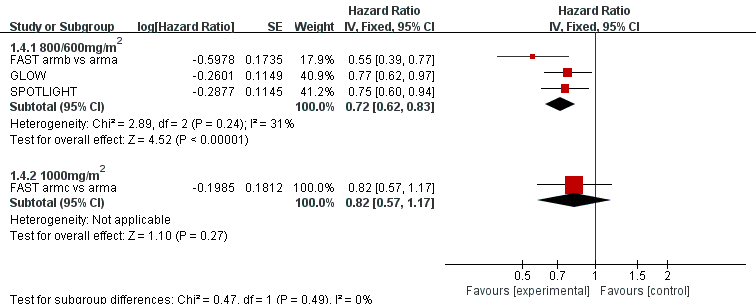
**

Abbreviations: The diamond indicates best estimate of the true (pooled) outcome (with width indicating 95% CI); HR, hazard ratio; experimental stands for zolbetuximab plus chemotherapy; control stands for chemotherapy alone. Since there is low heterogeneity, a fixed-effects model is used.

**eFigure. 2** Results of subgroup analysis of CLDN 18.2 expression for overall survival.

**
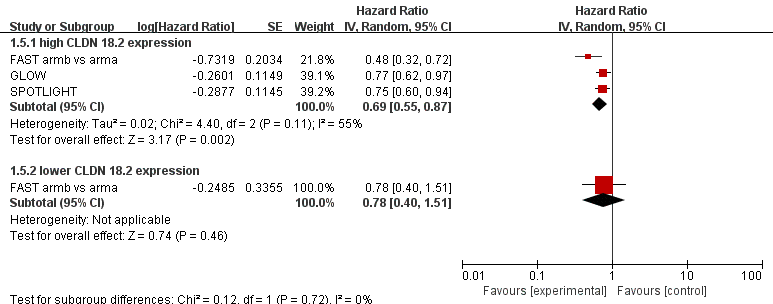
**

Abbreviations: The diamond indicates best estimate of the true (pooled) outcome (with width indicating 95% CI); HR, hazard ratio; experimental stands for zolbetuximab plus chemotherapy; control stands for chemotherapy alone. Since there is high heterogeneity, a random-effects model is used.

**eFigure. 3** Results of subgroup analysis of zolbetuximab dose for progression-free survival


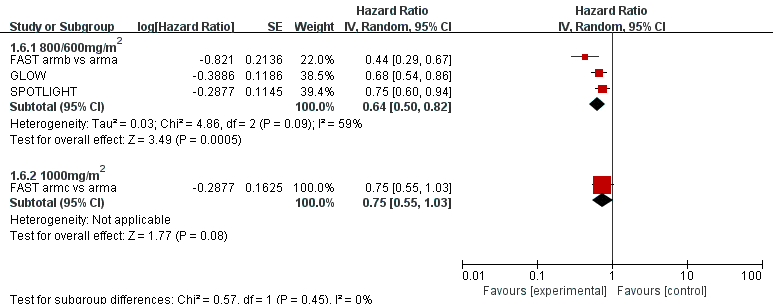


Abbreviations: The diamond indicates best estimate of the true (pooled) outcome (with width indicating 95% CI); HR, hazard ratio; experimental stands for zolbetuximab plus chemotherapy; control stands for chemotherapy alone. Since there is high heterogeneity, a random-effects model is used.

**eFigure. 4** Results of subgroup analysis of patients with CLDN 18.2 high expression for progression-free survival


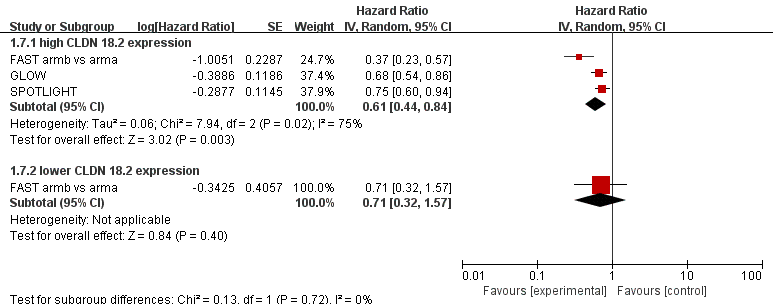


Abbreviations: The diamond indicates best estimate of the true (pooled) outcome (with width indicating 95% CI); HR, hazard ratio; experimental stands for zolbetuximab plus chemotherapy; control stands for chemotherapy alone. Since there is high heterogeneity, a random-effects model is used.

**eFigure. 5** Results for the effect of previous gastrectomy surgery in nausea


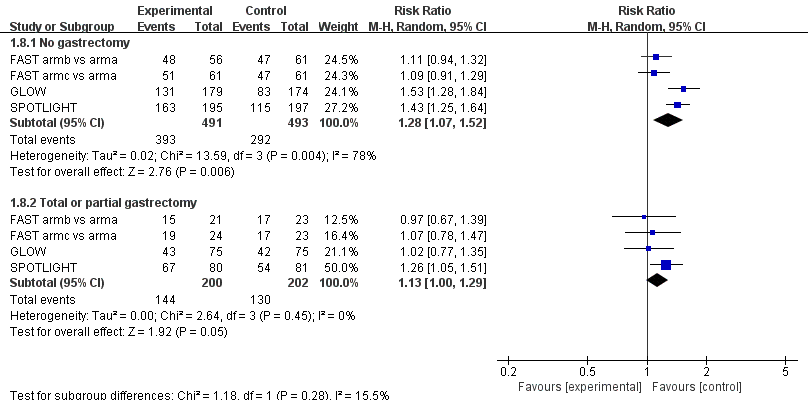


Abbreviations: The diamond indicates best estimate of the true (pooled) outcome (with width indicating 95% CI); RR, risk ratio; experimental stands for zolbetuximab plus chemotherapy; control stands for chemotherapy alone. Since there is high heterogeneity, a random-effects model is used.

**eFigure. 6** Results for the effect of previous gastrectomy surgery in voting


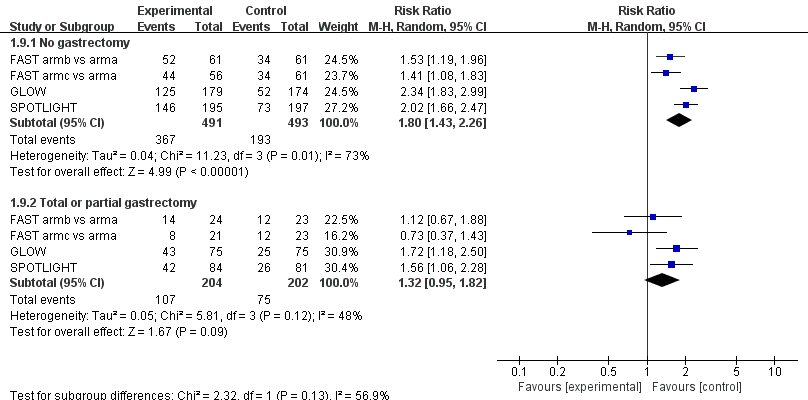


Abbreviations: The diamond indicates best estimate of the true (pooled) outcome (with width indicating 95% CI); RR, risk ratio; experimental stands for zolbetuximab plus chemotherapy; control stands for chemotherapy alone. Since there is high heterogeneity, a random-effects model is used.

**eFigure. 7** Results of subgroup analysis of patients with CLDN 18.2 high expression for overall survival.


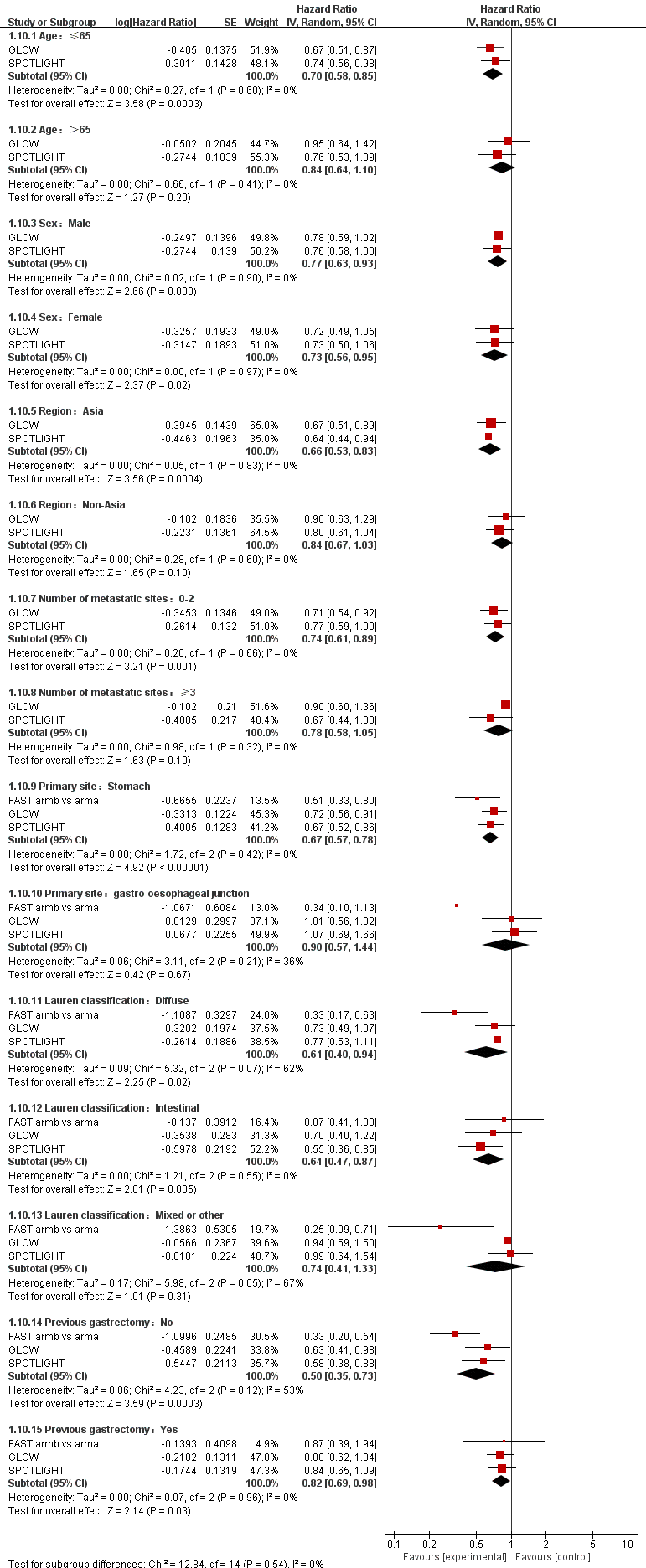


**eFigure. 8** Results of subgroup analysis of patients with CLDN 18.2 high expression for progression-free survival


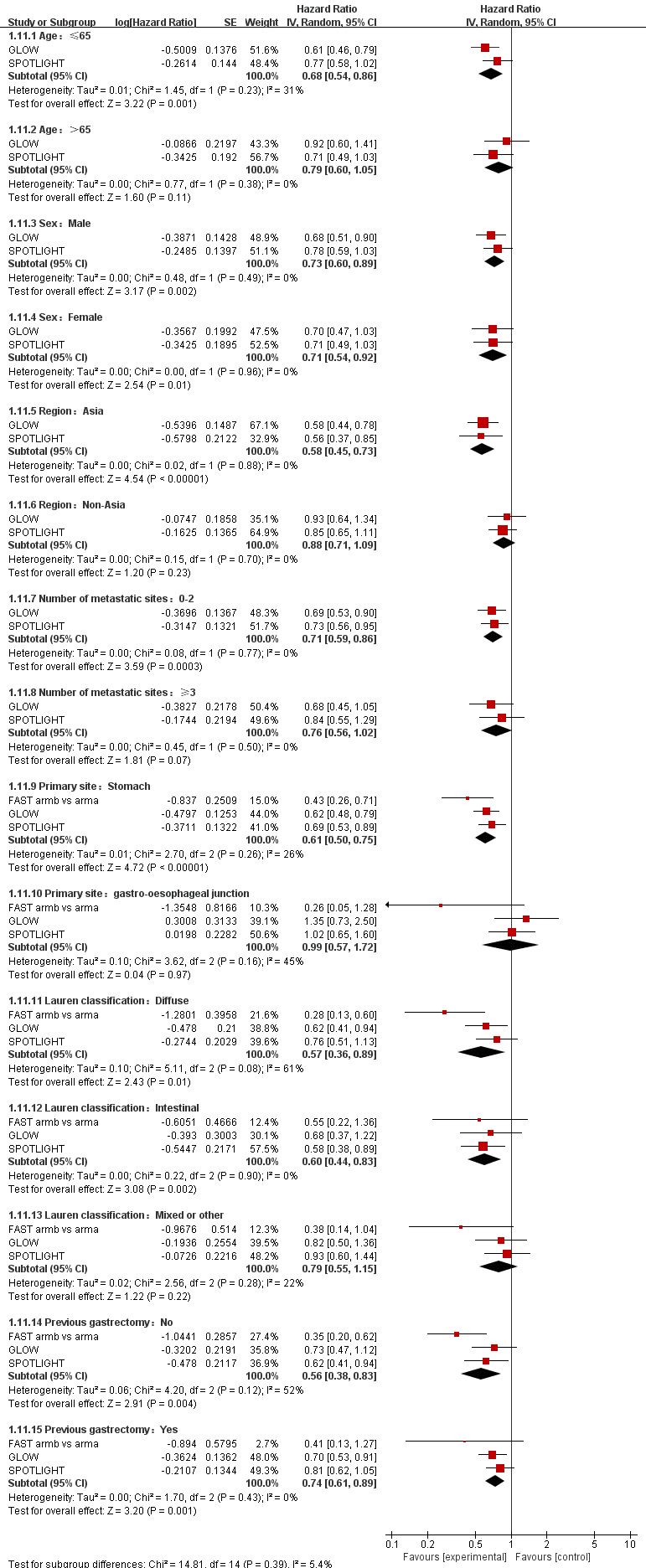


**eFigure. 9** Funnel plot of overall survival.


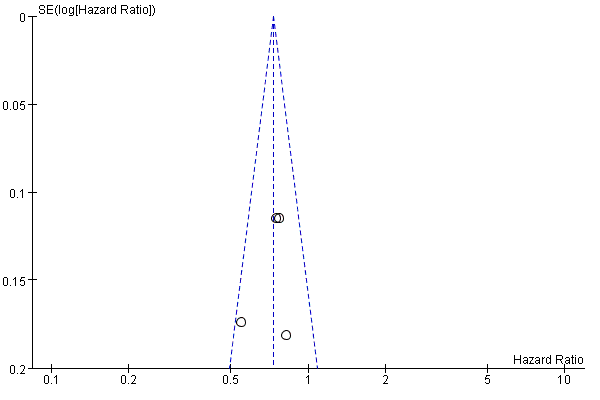


**eFigure. 10** Funnel plot of progression free survival.


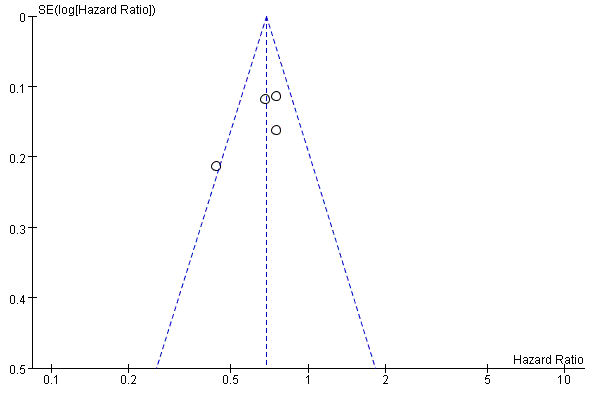


**eFigure. 11** Funnel plot of objective response rate


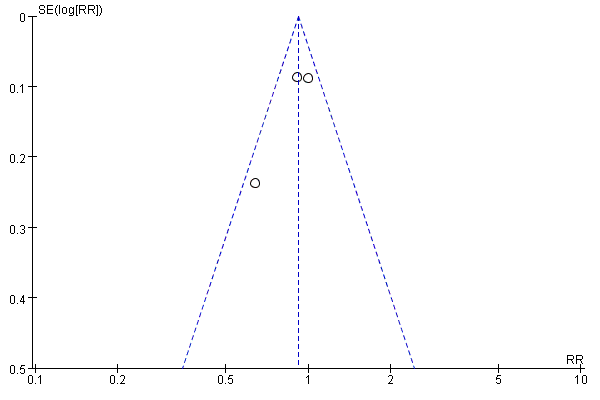


**eMethods.** Search Strategy

**PubMed < updated to 2023-06-10> Search Strategy (57)**

#1 "Stomach Neoplasms"[Mesh]

#2 Neoplasm, Stomach[Title/Abstract] OR Neoplasm, Stomach[Title/Abstract] OR Neoplasms, Stomach[Title/Abstract] OR Neoplasms, Stomach[Title/Abstract] OR Gastric Neoplasm[Title/Abstract] OR Gastric Neoplasm[Title/Abstract] OR Neoplasms, Gastric[Title/Abstract] OR Cancer of Stomach[Title/Abstract] OR Stomach Cancers[Title/Abstract] OR Gastric Cancer[Title/Abstract] OR Cancer, Gastric[Title/Abstract] OR Cancers, Gastric[Title/Abstract] OR Gastric Cancers[Title/Abstract] OR Stomach Cancer[Title/Abstract] OR Cancer, Stomach[Title/Abstract] OR Cancers, Stomach[Title/Abstract] OR Cancer of the Stomach[Title/Abstract] OR Gastric Cancer, Familial Diffuse[Title/Abstract] OR gastroesophageal junction adenocarcinoma[Title/Abstract] OR gastroesophageal junction cancer[Title/Abstract] OR gastroesophageal junction cancer[Title/Abstract] OR gastro- oesophageal junction adenocarcinoma[Title/Abstract]

#3 #1 OR #2

#4 "zolbetuximab" [Supplementary Concept

#5 IMAB362 OR IMAB-362 OR claudiximab OR zolbetuximab OR claudin 18.2 OR CLDN 18.2 OR claudin-18 isoform 2

#6 #4 OR #5

#7 #3 AND #6

**Embase < updated to 2023-06-10> Search Strategy（157）**

#1 'stomach tumor'/exp

#2 'neoplasm, stomach':ab,ti OR 'neoplasms, stomach':ab,ti OR 'gastric neoplasm':ab,ti OR 'neoplasms, gastric':ab,ti OR 'cancer of stomach':ab,ti OR 'stomach cancers':ab,ti OR 'gastric cancer':ab,ti OR 'cancer, gastric':ab,ti OR 'cancers, gastric':ab,ti OR 'gastric cancers':ab,ti OR 'stomach cancer':ab,ti OR 'cancer, stomach':ab,ti OR 'cancers, stomach':ab,ti OR 'cancer of the stomach':ab,ti OR 'gastric cancer, familial diffuse':ab,ti OR 'gastroesophageal junction adenocarcinoma':ab,ti OR 'gastroesophageal junction cancer':ab,ti OR 'gastro-oesophageal junction adenocarcinoma':ab,ti

#3 #1 OR #2

#4 'zolbetuximab'/exp

#5 imab362:ab,ti OR 'imab 362':ab,ti OR claudiximab:ab,ti OR zolbetuximab:ab,ti OR 'claudin 18.2':ab,ti OR 'cldn 18.2':ab,ti OR 'claudin-18 isoform 2':ab,ti

#6 #4 OR #5

#7 #3 AND #6

**Cochrane library < updated to 2023-06-10>（41）**

#1 MeSH descriptor: [Stomach Neoplasms] explode all trees

#2 (Neoplasm, Stomach OR Neoplasm, Stomach OR Neoplasms, Stomach OR Neoplasms, Stomach OR Gastric Neoplasm OR Gastric Neoplasm OR Neoplasms, Gastric OR Cancer of Stomach OR Stomach Cancers OR Gastric Cancer OR Cancer, Gastric OR Cancers, Gastric OR Gastric Cancers OR Stomach Cancer OR Cancer, Stomach OR Cancers, Stomach OR Cancer of the Stomach OR Gastric Cancer, Familial Diffuse OR gastroesophageal junction adenocarcinoma OR gastroesophageal junction cancer OR gastroesophageal junction cancer OR gastro-oesophageal junction adenocarcinoma):ti,ab,kw

#3 #1 OR #2

#4 (IMAB362 OR IMAB-362 OR claudiximab OR zolbetuximab OR claudin 18.2 OR CLDN 18.2 OR claudin-18 isoform 2):ti,ab,kw

#5 #3 AND #4
